# Safflower Yellow and Its Main Component HSYA Alleviate Diet-Induced Obesity in Mice: Possible Involvement of the Increased Antioxidant Enzymes in Liver and Adipose Tissue

**DOI:** 10.3389/fphar.2020.00482

**Published:** 2020-04-21

**Authors:** Kemin Yan, Xin Wang, Hui Pan, Linjie Wang, Hongbo Yang, Meijuan Liu, Huijuan Zhu, Fengying Gong

**Affiliations:** Key Laboratory of Endocrinology of National Health Commission, Department of Endocrinology, Peking Union Medical College Hospital, Chinese Academy of Medical Science and Peking Union Medical College, Beijing, China

**Keywords:** safflower yellow (SY), hydroxysafflor yellow A (HSYA), obesity, antioxidant enzymes, liver, adipose tissue

## Abstract

**Purpose:**

Oxidative stress plays an important role in the pathogenesis of obesity and its associated disorders. Safflower yellow (SY) and hydroxysafflor yellow A (HSYA), the natural compounds isolated from *Carthamus tinctorius L.*, has been found to possess antioxidative and anti-obesity properties. The purpose of the present study is to investigate whether SY and its main component HSYA alleviate obesity by the antioxidant effects.

**Methods:**

Diet-induced obese (DIO) mice were treated with 200 mg/kg/d SY or HSYA for 10 weeks. Body weight, fat mass, serum biochemical parameters and superoxide dismutase (SOD) activities were measured. Glucose and insulin tolerance tests were performed. The expression of antioxidant enzymes in liver and adipose tissue were measured. In vitro, H_2_O_2_-induced oxidative stress HepG2 cells and 3T3-L1 adipocytes were treated with SY and HSYA to investigate the direct effects of SY and HSYA on the expression of antioxidant enzymes.

**Results:**

SY and HSYA significantly decreased the body weight gain of DIO mice, and decreased fat mass to 57.8% and 61.6% of the control mice, respectively (*P* < 0.05). The parameters of glucose metabolism and liver function were improved after SY and HSYA treatment. The hepatic SOD activities and the mRNA levels of antioxidant enzymes in liver and adipose tissue of SY and HSYA treated mice were increased (*P* < 0.05). Meanwhile, the administration of SY and HSYA on the H_2_O_2_-induced oxidative stress HepG2 cells and adipocytes also increased the expression of the antioxidant factor and antioxidant enzymes to 1.2~3.3 folds of the control cells (*P* < 0.05).

**Conclusion:**

SY and its main component HSYA could significantly decrease the fat mass, and improve glucose metabolism and liver function in diet-induced obese mice. The beneficial effects of SY and HSYA on obesity and metabolism may be associated with the increased expression of antioxidant enzymes in liver and adipose tissue.

## Introduction

Obesity is a medical condition which results from the imbalance in energy metabolism, with excessive and abnormal fat accumulates in various organs and tissues. Fat accumulation has been reported to be closely correlated with the markers of systemic oxidative stress in humans and mice (Furukawa et al., 2004). Oxidative stress, mainly defined as the imbalance between the oxidative and anti-oxidative systems of the tissues and cells, plays an important role in the pathogenesis of various metabolic diseases (Rani et al., 2016). Oxidative stress in the adipose tissue has been reported to cause dysfunction of adipose tissue, which links to the development of obesity and its associated disorders, such as type 2 diabetes, cardiovascular diseases, and non-alcoholic fatty liver disease (NAFLD) (Bluher, 2009; Manna and Jain, 2015). Hepatic lipid accumulation and oxidative stress are major contributors in the pathophysiological mechanisms of NAFLD (Ashraf and Sheikh, 2015). Therefore, in order to prevent the development of these metabolic diseases, it is imperative to investigate novel therapeutic strategies to restore the normal equilibrium between oxidative and anti-oxidative processes. Antioxidant supplementation has been found to improve antioxidant-oxidant balance and the liver function in overweight or obese children and adolescents (Murer et al., 2014). In mouse models of inducible insulin resistance and obesity, antioxidant treatment has also been reported to protect against diabetes by improving glucose homeostasis and increasing insulin sensitivity (Straub et al., 2019).

*Carthamus tinctorius L.*, belongs to the Compositae or Asteraceae family, has been extensively used in traditional herbal medicine in China, Korea, Japan, and other Asian countries in treating various diseases (Zhang et al., 2016). Safflower yellow (SY) is the main constituent in the flower of *Carthamus tinctorius L.*, and hydroxysafflor yellow A (HSYA) is the major bioactive component of SY (Asgarpanah and Kazemivash, 2013; Fan et al., 2014). Recent studies have demonstrated that SY and HSYA possess multiple pharmacological functions, including antioxidation, anti-inflammation, anti-thrombosis, anti-coagulation, anti-tumor, anti-apoptosis, neuroprotection, and protection of endothelial cells (Fan et al., 2014; Zhang et al., 2016). The antioxidant effects of SY and HSYA have been reported in rat model of traumatic brain injury (Wang et al., 2016), HepG2 cells with oxidative damage (Ma et al., 2016), and pancreatic β-cells (Zhao et al., 2018). In our previous study, SY has been found to possess the anti-obesity effects (Zhu et al., 2016). In detail, SY significantly decreased the body fat mass, fasting blood glucose levels, and increased insulin sensitivity of diet-induced obese mice (Zhu et al., 2016). Similarly, the anti-obesity effects of SY and HSYA have also been demonstrated by other researchers. It was reported by Bao et al. that SY could reduce body weight and blood lipid levels in the mice fed with high fat diet (Bao et al., 2015). Liu et al. also found that HSYA could reduce body weight, fat accumulation, and insulin resistance in the high fat diet-fed mice by modulating the gut microbiota (Liu et al., 2018).

Oxidative stress is closely linked to the occurrence and development of obesity and its associated disorders. SY and its main component HSYA possess the antioxidant effects. However, whether the antioxidant effects of SY and HSYA involve in the mechanisms by which they reduce body weight and fat mass, as well as increase insulin sensitivity still remains unclear. The hypothesis of the present study is that SY and HSYA play the anti-obesity role by its antioxidant effects. Therefore, experiments were conducted on diet-induced obese (DIO) mice and the cellular models of oxidative stress damage to investigate the impacts of SY and its main component HSYA on alleviating obesity and the association with the antioxidant effects in liver, adipose tissue, and cells.

## Materials and Methods

### Preparation of SY and HSYA

SY used in our experiments was isolated from the water-soluble extracts of the flower of *Carthamus tinctorius L.* (produced in Tacheng City, Tacheng Prefecture, Xinjiang Uyghur Autonomous Region) by Prof. Ming Jin (Beijing An Zhen Hospital, Capital Medical University). SY is composed of a variety of chalcone mixtures. Among them, HYSA is the main active component. The concentration of HSYA in the SY is 43.5 g/L (determined by spectrophotometry). The purity of HSYA in the SY is 75.56% (determined by HPLC), which refers to the purity percentage of the main peak calculated by the peak area normalization method. The chemical compound HSYA (purity 98.43%) was purchased from Chengdu Herbpurify Co., Ltd. (Sichuan, China). The HPLC analysis of SY and HSYA were presented in the supplementary materials. SY and HSYA were dissolved in deionized water.

### Animals

Male 7-week-old C57BL/6J mice were purchased from Beijing Vital River Laboratory Animal Technology Co., Ltd. (Beijing, China), and housed in a standard 12-h light/dark cycle with free access to food and water. After 1 week of acclimation, mice were randomly assigned to a standard food (SF; 10% kcal fat, n=20; H10010, Beijing HFK Bioscience Co., Ltd., Beijing, China) group and a high-fat diet (HFD; 45% kcal fat, n=30; H10045, Beijing HFK Bioscience Co., Ltd., Beijing, China) group. The compositions of the experimental diets are shown in **Table S1**. Ten weeks later, mice fed with SF were divided into SF-Saline group (n=10) and SF-SY group (n=10), and mice fed with HFD were weighed and divided into HFD-Saline group (n=10), HFD-SY group (n=10), and HFD-HSYA group (n=10). Mice in the SY and HSYA intervention group were intraperitoneally injected with 200 mg/kg/d SY or HSYA for ten weeks, and mice in SF-Saline and HFD-Saline groups were intraperitoneally injected with equal volume of saline. Body weight was recorded twice a week, and food intake was recorded weekly. The animal experiment protocols were approved by the ethics committee of Peking Union Medical College Hospital.

### Intraperitoneal Glucose Tolerance Test (IPGTT), Intraperitoneal Insulin Tolerance Test (IPITT), and Sample Collection

Ten weeks after SY and HSYA intervention, IPGTT and IPITT were performed. In IPGTT, mice were overnight fasted and 50% glucose (2 g/kg) was administrated intraperitoneally. In IPITT, mice were morning fasted for 5 h and insulin (0.72 IU/kg, Novolin R, Novo Nordisk, Denmark) was administered intraperitoneally. Blood glucose levels were measured from the tail at 0, 30, 60, 90, and 120 min after the administration. Area under the curve (AUC) was calculated by trapezoidal integration. There were two mice in SF-SY group, one in HFD-SY group and one in HFD-HSYA group died in IPITT due to hypoglycemia. Two days after IPITT, mice were starved overnight and anesthetized. Blood samples were collected and centrifuged at 3,000 rpm for 10 min at 4°C for serum collection. Liver tissue and white adipose tissue (WAT), including subcutaneous WAT (sWAT), epididymal WAT (eWAT), and perirenal WAT, were harvested, snap frozen in liquid nitrogen, and stored at −80°C. Part of the liver tissue was fixed in 10% formalin.

### Measurements of Biochemical Parameters, Superoxide Dismutase (SOD) Activities, and Malondialdehyde (MDA)

Serum biochemical parameters were measured by routine automated laboratory methods. Insulin levels were measured by a commercial ELISA kit (MEA448Mu, Wuhan USCN Business Co., Ltd., Wuhan, China) according to the manufacturer’s instruction. The intra-assay coefficient of variation was 4.0%. Homeostasis model assessment of insulin resistance (HOMA-IR) was calculated as described previously (Yan et al., 2017). SOD activities and MDA levels in tissues and serum were measured by the SOD assay kit (A001-3, Nanjing Jiancheng Bioengineering Institute, Nanjing, China) and MDA assay kit (A003-1, Nanjing Jiancheng Bioengineering Institute, Nanjing, China), respectively, according to the instruction manual.

### Liver Histologic Analysis and Triglyceride (TG) Contents Measurement

Liver tissue fixed in 10% formalin was dehydrated and embedded in paraffin. Sections (3 μm) were stained with hematoxylin and eosin (H&E) by standard procedures. Images were obtained using a digital camera (Nikon DS-U3, Japan). Liver TG contents were measured by a triglyceride assay kit (A110-1-1, Nanjing Jiancheng Bioengineering Institute, Nanjing, China) according to the instruction manual.

### Cell Culture

HepG2 cells were purchased from the Cell Resource Center, Institute of Basic Medical Sciences, Chinese Academy of Medical Sciences (Beijing, China), and maintained in Minimum Essential Medium with Earle’s Balanced Salts (SH30024.01, HyClone, Logan, USA) with 10% (v/v) fetal bovine serum (FBS), 0.1 mM non-essential amino acids, 1 mM sodium pyruvate, 100 U/mL penicillin and 100 µg/mL streptomycin at 37°C in a 5% CO_2_ incubator. HepG2 cells were pretreated with 10, 50, and 100 mg/L SY for 24 h, and then treated with 20 μM hydrogen peroxide (H_2_O_2_) solution for 24 h, following the total RNA extraction.

3T3-L1 preadipocytes were obtained from the Cell Resource Center, Institute of Basic Medical Sciences, Chinese Academy of Medical Sciences (Beijing, China), and cultured in Dulbecco’s Modified Eagle Medium (DMEM, SH30022.01, HyClone, Logan, USA) with 10% (v/v) bovine calf serum, 100 U/ml penicillin and 100 µg/ml streptomycin at 37°C in a 5% CO_2_ incubator. Two days after confluence, 3T3-L1 preadipocytes were induced to differentiation in DMEM with 10% FBS, 10 μg/ml insulin, 0.5 mM 3-isobutyl-1-methylxanthine, and 10 μM dexamethasone for 48 h, and then in DMEM containing 10% (v/v) FBS and 10 μg/ml insulin for 48 h. After that, the medium was replaced every other day with DMEM containing 10% (v/v) FBS. Six days later, the differentiated 3T3-L1 adipocytes were pretreated with 10, 50, and 100 mg/L SY or HSYA for 24 h, and then administrated with 200 μM H_2_O_2_ solution for 24 h, following the total RNA extraction.

### RT-qPCR

Total RNA was extracted from the liver tissue, adipose tissue, HepG2 cells, and 3T3-L1 adipocytes using Total RNA Kit II (R6934, Omega Biotek, USA) according to the instruction manual. Reverse transcription was performed using the PrimeScript™ RT reagent Kit with gDNA Eraser (RR047A, TaKaRa, Japan). qPCR analysis was performed in ABI7500 PCR system (Applied Biosystems, San Francisco, CA, USA) using TB Green^®^ Premix Ex Taq™ II (RR820A, TaKaRa, Japan) to measure the expression of nuclear factor erythroid 2-related factor 2 (Nrf2) and antioxidant enzymes, including SOD1, glutamate-cysteine ligase catalytic subunit (GCLC), NAD(P)H dehydrogenase (quinone 1) (Nqo1), catalase (CAT), and heme oxygenase-1 (HO-1). Peptidylprolyl isomerase A (PPIA) or GAPDH was used for normalization. The relative expression of each target gene was calculated by the 2^−ΔΔCt^ method (Livak and Schmittgen, 2001). The primers used in RT-qPCR were listed in the **Table S2**.

### Statistical Analysis

All data were expressed as mean ± standard deviation (SD). Statistical analysis was performed by SPSS software (version 22.0 for Windows, SPSS Inc., Chicago, IL, USA). The univariate analysis of variance (ANOVA) was used for data analysis, with Bonferroni *post hoc* test for multiple comparison. The Kruskal-Wallis test was used if the ANOVA was inapplicable. *P* < 0.05 was considered statistically significant.

## Results

### SY and HSYA Decreased Body Weight Gain, WAT Mass, and WAT Percentage in DIO Mice

**Figure 1:**
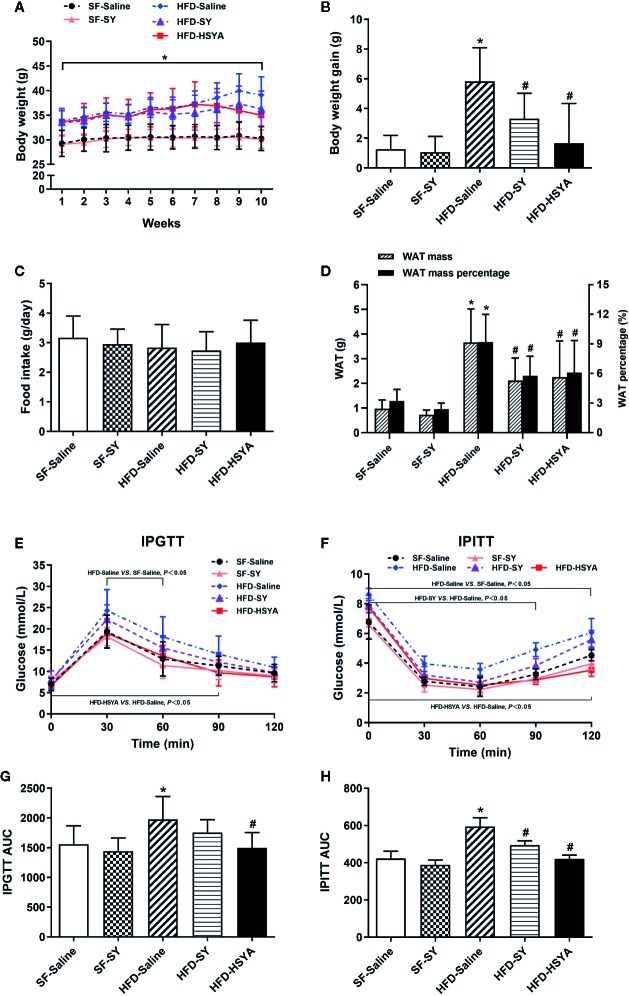
Effects of safflower yellow (SY) and hydroxysafflor yellow A (HSYA) on body weight, food intake, fat mass, glucose tolerance test, and insulin tolerance test in mice. Mice were intraperitoneally injected with 200 mg/kg/d SY or HSYA for 10 weeks. Body weight **(A)** was recorded, and the body weight gains **(B)** were calculated at the end of the experiment. Food intake **(C)** was also recorded. Ten weeks after intervention, white adipose tissue (WAT), including subcutaneous WAT, epididymal WAT and perirenal WAT, were obtained and weighed **(D)**. The WAT percentage was calculated by the percentage of body weight occupied by the WAT mass **(D)**. The intraperitoneal glucose tolerance test (IPGTT, **E**) and the intraperitoneal insulin tolerance test (IPITT, **F**) and were performed. Areas under the curve (AUCs) of the IPGTT and IPITT were calculated **(G**, **H)**. The data are represented as the mean ± SD. ^*^ *P* < 0.05 vs. SF-Saline, ^#^ *P* < 0.05 vs. HFD-Saline. (n=10 in the SF-Saline group, n=8 in the SF-SY group, n=10 in the HFD-Saline group, n=9 in the HFD-SY group, n=9 in the HFD-HSYA group).

As presented in **Figure 1**, the body weight and body weight gain of the DIO mice were significantly increased, and the WAT mass and WAT percentage of DIO mice were also significantly increased to 3.8- and 2.9-folds of the SF-Saline group (*P* < 0.05). After SY treatment for 10 weeks, the body weight gain, WAT mass and WAT percentage of the DIO mice significantly decreased to 56.8%, 57.8%, and 62.7% of the HFD-Saline group (*P* < 0.05). Similarly, after HSYA treatment, the body weight gain, WAT mass, and WAT percentage of the DIO mice also remarkably decreased to 28.7%, 61.6%, and 66.3% of the HFD-Saline group (*P* < 0.05). However, SY administration had no effect on the body weight gain and WAT mass in the lean mice fed with SF. Both SY and HSYA administration had no effect on food intake of the lean and DIO mice. In order to eliminate the effect of daily intraperitoneal injection-induced chronic inflammation, a preliminary experiment for evaluating the effects of SY and HSYA by intragastric administration was being performed. The preliminary results showed that SY and HSYA could also decrease the body weight and body weight gain of the mice by intragastric administration (**Figures S1A, B**, *P* < 0.05). There was also no effect of SY and HSYA on food intake by intragastric administration (**Figure S1C**).

### SY and HSYA Improved Glucose Metabolism and Liver Function in DIO Mice

The DIO mice showed abnormal glucose metabolism. When compared with the SF-Saline group, the blood glucose levels of DIO mice in HFD-Saline group were remarkably increased in IPGTT and IPITT (**Figures 1E, F**, *P* < 0.05), and the AUC of IPGTT and IPITT of DIO mice also significantly increased by 26.8% and 40.5% (**Figures 1G, H**, *P* < 0.05). Meanwhile, serum levels of fasting blood glucose (FBG), total cholesterol, and high-sensitivity C-reactive protein of the DIO mice were all significantly increased (**Table 1**, *P* < 0.05). It was observed that SY and HSYA could improve glucose metabolism in the DIO mice. In the HFD-SY group, the blood glucose levels in IPITT were decreased, and the AUC of IPITT showed a 16.6% decrease when compared with the HFD-Saline group (**Figures 1F, H**, *P* < 0.05). FBG and HOMA-IR of the SY treated mice were also decreased by 18.3% and 32.2%, respectively (**Table 1**, *P* < 0.05). Consistently, in the HFD-HSYA group, the blood glucose levels in both IPGTT and IPITT were significantly decreased (**Figures 1E, F**, *P* < 0.05), the AUC of IPGTT and IPITT were also decreased by 24.1% and 29.1% (**Figures 1G, H**, *P* < 0.05), and the FBG and HOMA-IR were decreased by 15.7% and 34.5% (**Table 1**, *P* < 0.05) when compared with the HFD-Saline group. However, SY had no effect on glucose metabolism of the lean mice.

**Table 1:** Serum biochemical parameters of the mice.

	SF-Saline (n=10)	SF-SY (n=8)	HFD-Saline (n=10)	HFD-SY (n=9)	HFD-HSYA (n=9)
**FBG (mmol/L)**	7.58 ± 2.37	8.35 ± 1.41	11.62 ± 1.61^*^	9.49 ± 2.25^#^	9.80 ± 1.89^#^
**Insulin (ng/mL)**	1.60 ± 0.50	1.10 ± 0.48	1.20 ± 0.42	1.02 ± 0.35	0.93 ± 0.34
**HOMA-IR**	14.83 ± 4.04	11.92 ± 5.49	18.16 ± 8.78	12.31 ± 4.52^#^	11.89 ± 5.28^#^
**TC (mmol/L)**	4.02 ± 0.32	3.83 ± 0.36	4.71 ± 0.78^*^	4.70 ± 0.44	4.54 ± 0.90
**TG (mmol/L)**	0.30 ± 0.21	0.24 ± 0.07	0.28 ± 0.07	0.32 ± 0.14	0.30 ± 0.22
**LDL-c (mmol/L)**	0.42 ± 0.06	0.46 ± 0.11	0.57 ± 0.15	0.59 ± 0.15	0.55 ± 0.18
**HDL-c (mmol/L)**	1.91 ± 0.12	1.75 ± 0.13	1.95 ± 0.23	1.92 ± 0.18	1.84 ± 0.15
**hsCRP (mg/L)**	0.08 ± 0.03	0.07 ± 0.03	0.14 ± 0.03^*^	0.11 ± 0.03	0.09 ± 0.02
**Cr (μmol/L)**	7.16 ± 1.61	7.08 ± 0.79	7.77 ± 1.55	5.84 ± 1.12^#^	7.23 ± 1.15
**ALT (U/L)**	15.04 ± 1.22	14.23 ± 2.88	33.09 ± 9.20^*^	23.02 ± 8.15^#^	23.90 ± 10.06^#^
**AST (U/L)**	99.15 ± 16.46	91.80 ± 11.35	114.29 ± 27.07	97.29 ± 9.05	110.99 ± 18.48

Values are mean ± SD. ALT, alanine aminotransferase; AST, aspartate aminotransferase; Cr, creatinine; FBG, fasting blood glucose; HDL-, high density lipoprotein-cholesterol; HOMA-IR, homeostasis model assessment of insulin resistance; hsCRP, high-sensitivity C reactive protein; LDL-c, low density lipoprotein-cholesterol; TC, total cholesterol; TG, triglycerides. *P < 0.05 vs. SF-Saline; ^#^P < 0.05 vs. HFD-Saline.

**Figure 2:**
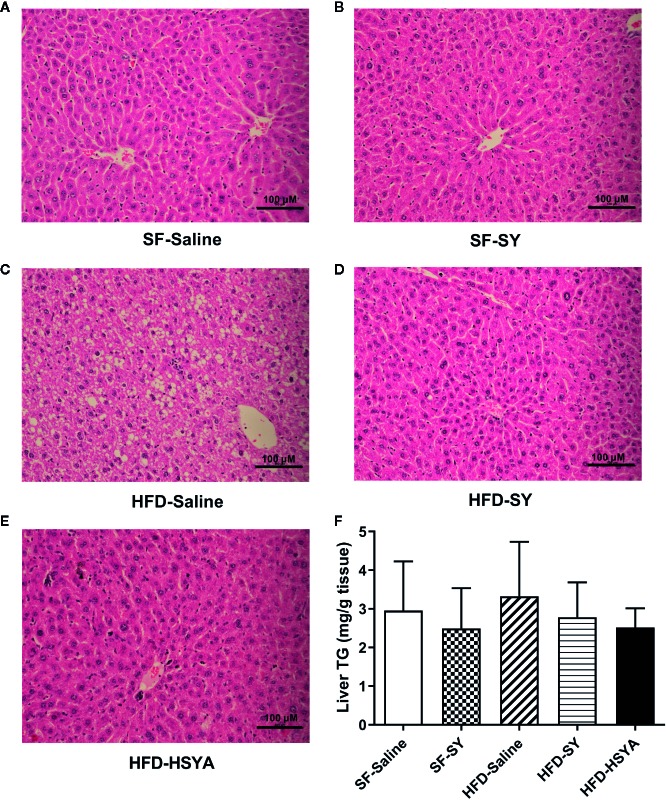
Hematoxylin-Eosin (H&E) stained sections of liver tissues and liver triglyceride (TG) contents of the mice. Mice were intraperitoneally injected with 200 mg/kg/d safflower yellow (SY) or hydroxysafflor yellow A (HSYA) for 10 weeks. Liver tissue was fixed in 10% formalin, and then dehydrated and embedded in paraffin. Sections (3 μm) were stained with H&E. Images of the representative sections in the SF-Saline group **(A)**, SF-SY group **(B)**, HFD-Saline group **(C)**, HFD-SY group **(D)**, and HFD-HSYA group **(E)** at 200× magnification (scale bars, 100 μm) were obtained using a digital camera. Liver TG contents were measured by a triglyceride assay kit **(F)**. The data are represented as the mean ± SD. (n=10 in the SF-Saline group, n=8 in the SF-SY group, n=10 in the HFD-Saline group, n=9 in the HFD-SY group, n=9 in the HFD-HSYA group).

Serum ALT levels of the DIO obese mice were increased to 2.2-folds of that in SF-Saline group, and significantly decreased by 30.4% and 27.8%, respectively, after SY and HSYA administration (**Table 1**, *P* < 0.05). As presented in **Figure 2** and supplementary **Figure S2**, there were many lipid droplets in the H&E and Oil Red O staining of liver tissue in the HFD-Saline group, companied by an increasing trend of the liver TG contents. By contrast, there were less lipid drops in the SY and HSYA treated DIO mice, and TG contents measurement also revealed the tendency to decrease. SY also showed no effect on liver function of the lean mice.

### SY and HSYA Increased the SOD Activities in Liver Tissue of DIO Mice

**Figure 3:**
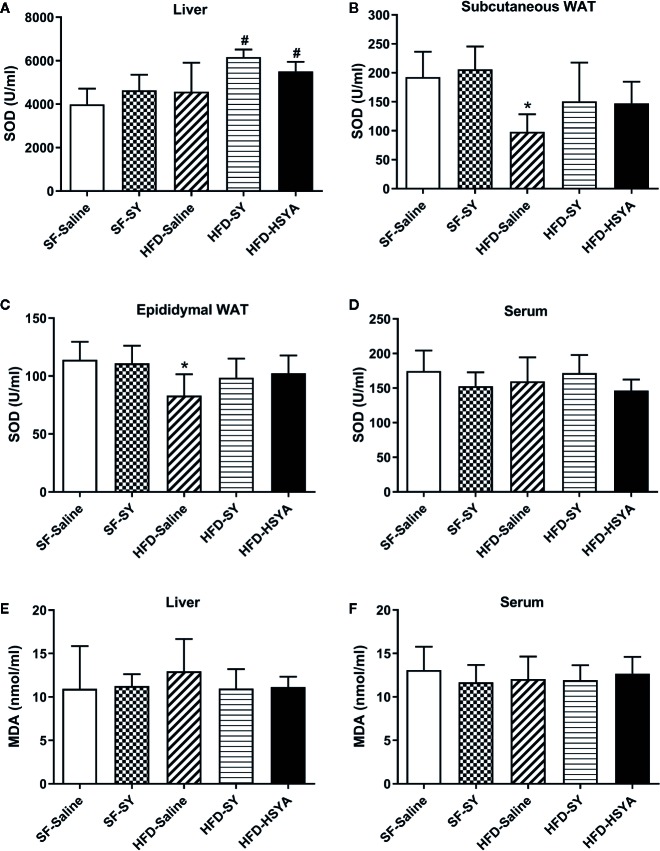
Effects of safflower yellow (SY) and hydroxysafflor yellow A (HSYA) on superoxide dismutase (SOD) activities and malondialdehyde (MDA) levels in mice. Mice were intraperitoneally injected with 200 mg/kg/d SY or HSYA for 10 weeks. Blood samples were collected and centrifuged for serum collection. Liver tissue, subcutaneous white adipose tissue (WAT) and epididymal WAT were obtained. SOD activities of liver **(A)**, adipose tissue **(B, C)**, and serum **(D)** were measured by SOD assay kit. MDA levels in liver **(E)** and serum **(F)** were measured by MDA assay kit. The data are represented as the mean ± SD. ^*^ *P* < 0.05 vs. SF-Saline, ^#^ *P* < 0.05 vs. HFD-Saline. (n=10 in the SF-Saline group, n=8 in the SF-SY group, n=10 in the HFD-Saline group, n=9 in the HFD-SY group, n=9 in the HFD-HSYA group).

Since SY and HSYA have been reported to possess antioxidant effects, SOD activities of serum, liver and white adipose tissue were measured. The results showed that the SOD activities in the liver tissue of SY and HSYA treated DIO mice were increased by 34.8% and 20.3%, respectively, when compared with the HFD-Saline group (**Figure 3A**, *P* < 0.05). In both sWAT and eWAT, the SOD activities of DIO mice were reduced to 50.9% and 72.8%, respectively, of that in the SF-Saline group (**Figures 3B, C**, *P* < 0.05), and showed the trend to increase after SY and HSYA treatment. However, there was no change in the serum SOD activities after SY and HSYA treatment in DIO mice. There was only a decreasing trend in serum levels of tumor necrosis factor α (TNFα) after SY intervention, but no statistical difference (p=0.067, **Figure S3**). SY also had no effect on the SOD activities of both liver and adipose tissue of the lean mice. There was also no significant difference in MDA levels among those groups.

### SY and HSYA Increased the Expression of Antioxidant Enzymes in Liver Tissue and Adipose Tissue of DIO Mice

**Figure 4:**
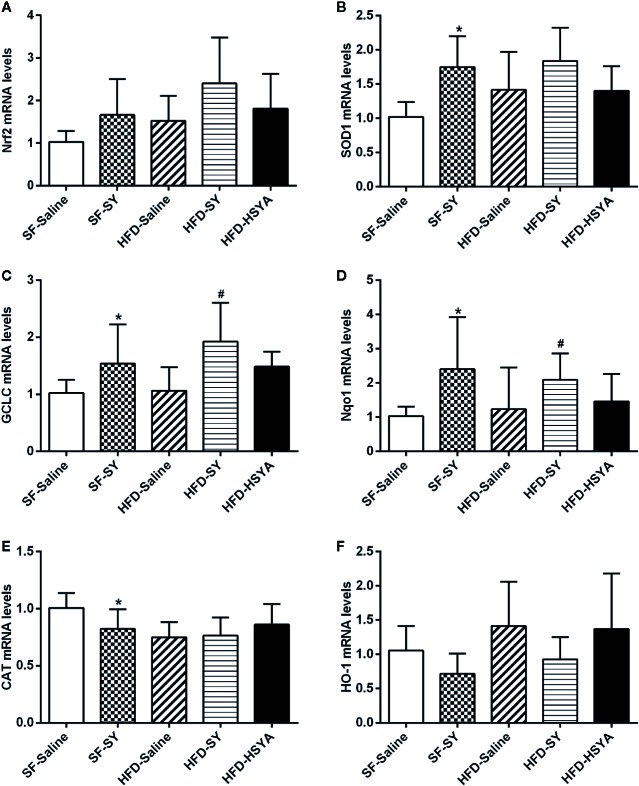
Effects of safflower yellow (SY) and hydroxysafflor yellow A (HSYA) on the expression of Nrf2 and antioxidant enzymes in the liver tissues of the mice. Mice were intraperitoneally injected with 200 mg/kg/d SY or HSYA for 10 weeks. Liver tissue was obtained and total RNA was extracted. The messenger RNA (mRNA) levels of nuclear factor erythroid 2-related factor 2 (Nrf2) and antioxidant enzymes, including superoxide dismutase 1 (SOD1), glutamate-cysteine ligase catalytic subunit (GCLC), NAD(P)H dehydrogenase (quinone 1) (Nqo1), catalase (CAT), and heme oxygenase-1 (HO-1), were determined by RT-qPCR analysis **(A**–**F)**. The data are represented as the mean ± SD. ^*^ *P* < 0.05 vs. SF-Saline, ^#^ *P* < 0.05 vs. HFD-Saline. (n=8 in each group).

As shown in **Figure 4**, SY increased the mRNA levels of SOD1, GCLC, and Nqo1 in liver tissue of the lean mice to 1.7-, 1.5-, and 2.3-folds of the SF-Saline group (*P* < 0.05). In DIO mice, SY could also increase the mRNA levels of GCLC and Nqo1 to 1.8- and 1.7-folds in liver tissue when compared with the HFD-Saline group (*P* < 0.05). However, there was a decrease in the mRNA levels of CAT in liver tissue of the lean mice after SY treatment (*P* < 0.05).

**Figure 5:**
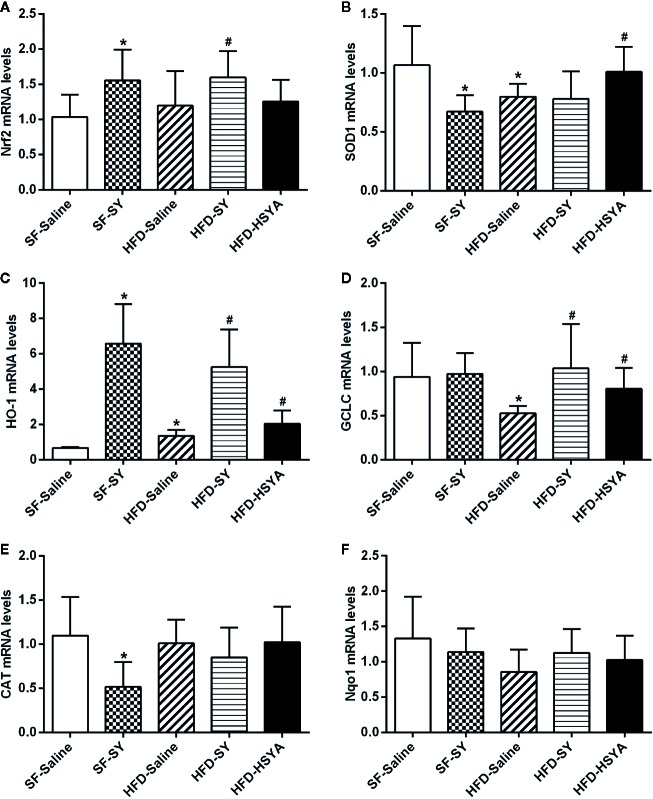
Effects of safflower yellow (SY) and hydroxysafflor yellow A (HSYA) on the expression of Nrf2 and antioxidant enzymes in the epididymal adipose tissues of the mice. Mice were intraperitoneally injected with 200 mg/kg/d SY or HSYA for 10 weeks. The epididymal adipose tissue was obtained and total RNA was extracted. The messenger RNA (mRNA) levels of nuclear factor erythroid 2-related factor 2 (Nrf2) and antioxidant enzymes, including superoxide dismutase 1 (SOD1), heme oxygenase-1 (HO-1), glutamate-cysteine ligase catalytic subunit (GCLC), catalase (CAT), and NAD(P)H dehydrogenase (quinone 1) (Nqo1) were determined by RT-qPCR analysis **(A**–**F)**. The data are represented as the mean ± SD. ^*^ *P* < 0.05 vs. SF-Saline, ^#^ *P* < 0.05 vs. HFD-Saline. (n=8 in each group).

As to the eWAT, there was impairment in the expression of antioxidant enzymes in DIO mice. The mRNA levels of SOD1 and GCLC in eWAT of the HFD-Saline group significantly decreased by 25.0% and 44.0% when compared with the SF-Saline group (**Figures 5B, D**, *P* < 0.05), while the HO-1 mRNA levels increased to 2.0-folds of the SF-Saline group (**Figure 5C**, *P* < 0.05). SY and HSYA treatment obviously increased the expression of antioxidant factor and enzymes in the eWAT of DIO mice. In the SY-treated obese mice, the mRNA levels of the antioxidant factor Nrf2 and the antioxidant enzymes HO-1 and GCLC were obviously increased to 1.3-, 3.9-, and 2.0-folds of that in HFD-Saline group (**Figures 5A, C, D**, *P* < 0.05). Meanwhile, the mRNA levels of SOD1, HO-1, and GCLC in eWAT of DIO mice significantly increased by 20.9%, 50.8%, and 53.0% after HSYA treatment in comparison with HFD-Saline group (**Figures 5B–D**, *P* < 0.05). Besides, in the SY-treated lean mice, the mRNA levels of Nrf2 and HO-1 in the eWAT significantly increased to 1.5- and 9.8-folds of that in SF-Saline group (**Figures 5A, C**, *P* < 0.05), while there was a decrease in the mRNA levels of SOD1 and CAT (**Figures 5B, E**, *P* < 0.05). However, there was no significant change in the mRNA levels of these antioxidant enzymes in the sWAT after SY and HSYA treatment (**Figure S4**).

### SY Increased the Expression of Nrf2 and Antioxidant Enzymes in HepG2 Cells

**Figure 6:**
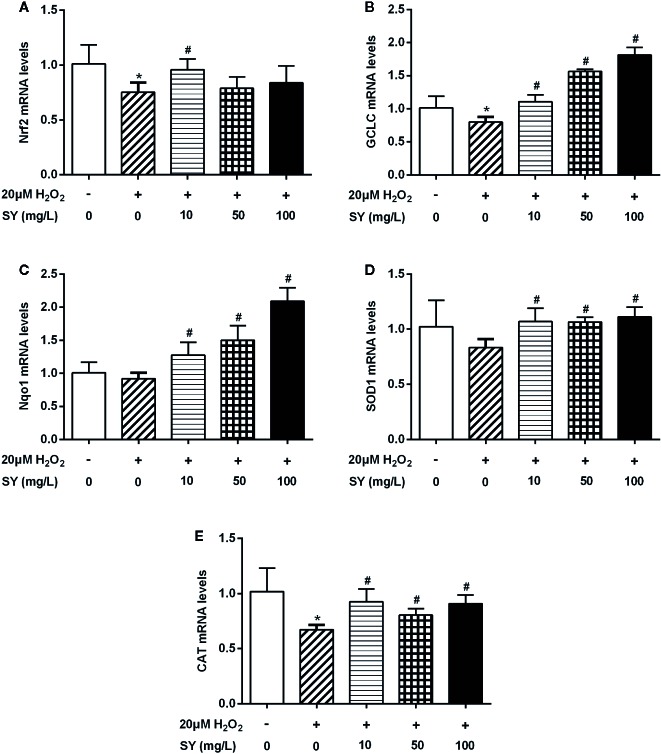
Effects of safflower yellow (SY) on the expression of Nrf2 and antioxidant enzymes in HepG2 cells. HepG2 cells were plated in 24-well plates and pretreated with 10, 50, and 100 mg/L SY for 24 h, and then treated with 20 μM hydrogen peroxide (H_2_O_2_) solution for 24 h. Then, cells were lysed for total RNA extraction. RT-qPCR analysis was performed to measure the mRNA levels of nuclear factor erythroid 2-related factor 2 (Nrf2) and antioxidant enzymes, including glutamate-cysteine ligase catalytic subunit (GCLC), NAD(P)H dehydrogenase (quinone 1) (Nqo1), superoxide dismutase 1 (SOD1), and catalase (CAT) **(A**–**E)**. The data are represented as the mean ± SD of three separate wells. ^*^ *P* < 0.05 vs. control cells (without H_2_O_2_ and SY), ^#^ *P* < 0.05 vs. H_2_O_2_ group (with 20 μM H_2_O_2_).

HepG2 cells were treated with H_2_O_2_ solution to simulate a state of oxidative stress with impairment in the expression of Nrf2 and antioxidant enzymes. The effect of H_2_O_2_ on cell viability of HepG2 cells was examined by MTT assay. There was no significant decrease in cell viability after 20 μM H_2_O_2_ treated for 24 h (**Figure S5A**). As presented in **Figure 6**, the mRNA levels of Nrf2, GCLC and CAT in H_2_O_2_ treated HepG2 cells were decreased by 25.6%, 21.2%, and 34.0% in comparison with control cells (*P* < 0.05), suggesting that the oxidative stress cell model was successfully established. SY treatment presented a protective effect in HepG2 cells by restoring the antioxidant capacity. After 10 mg/L SY administration, Nrf2 mRNA levels increased to 1.3 folds of H_2_O_2_ treated group (**Figure 6A**, *P* < 0.05). SY also increased the mRNA levels of GCLC and Nqo1 in a dose-dependent manner. After 10, 50, and 100 mg/L SY treatment, GCLC mRNA levels significantly increased to 1.4-, 2.0-, and 2.3-folds of H_2_O_2_ treated group, and Nqo1 mRNA levels significantly increased to 1.4-, 1.6-, and 2.3-folds of H_2_O_2_ treated group (**Figures 6B, C**, *P* < 0.05). Meanwhile, the mRNA levels of SOD1 and CAT were also increased to 1.2~1.4-folds after 10, 50, and 100 mg/L SY treatment (**Figures 6D, E**, *P* < 0.05).

### SY and HSYA Increased the Expression of Nrf2 and Antioxidant Enzymes in 3T3-L1 Adipocytes

**Figure 7:**
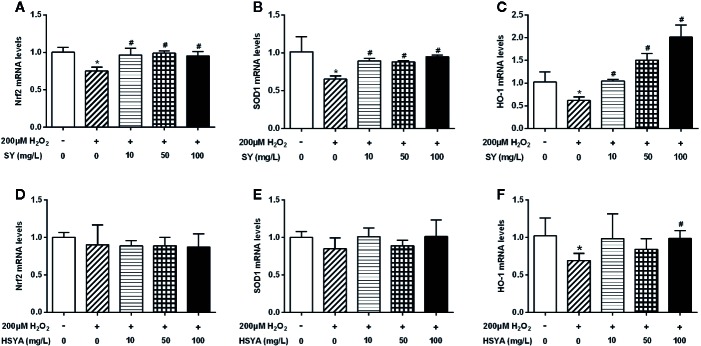
Effects of safflower yellow (SY) and hydroxysafflor yellow A (HSYA) on the expression of Nrf2 and antioxidant enzymes in 3T3-L1 adipocytes. 3T3-L1 preadipocytes were plated in 24-well plates and induced to differentiation. The differentiated 3T3-L1 adipocytes were pretreated with 10, 50, and 100 mg/L SY or HSYA for 24 h, and then administrated with 200 μM H_2_O_2_ solution for 24 h. Then, cells were lysed for total RNA extraction. RT-qPCR analysis was performed to measure the changes in the messenger RNA (mRNA) levels of nuclear factor erythroid 2-related factor 2 (Nrf2) and antioxidant enzymes, including superoxide dismutase 1 (SOD1) and heme oxygenase-1 (HO-1), after SY **(A**–**C)** or HSYA **(D**–**F)** administration. The data are represented as the mean ± SD of three separate wells. ^*^ *P* < 0.05 vs. control cells (without H_2_O_2_ and SY/HSYA), ^#^ *P* < 0.05 vs. H_2_O_2_ group (with 200 μM H_2_O_2_).

There was no significant decrease in cell viability of 3T3-L1 adipocytes after 200 μM H_2_O_2_ treated for 24h (**Figure S5B**). H_2_O_2_ solution treatment also simulate a state of oxidative stress in 3T3-L1 adipocytes. The mRNA levels of Nrf2, SOD1, and HO-1 in H_2_O_2_ treated 3T3-L1 adipocytes were decreased by 25.1%, 35.7%, and 39.7% when compared with control cells (**Figure 7**, *P* < 0.05). SY could also restore the antioxidant capacity of the H_2_O_2_-induced oxidative stress damage 3T3-L1 adipocytes. After 10, 50, and 100 mg/L SY intervention, the mRNA levels of Nrf2 and SOD1 were significantly increased to 1.3~1.5-folds of that in H_2_O_2_ treated group (**Figures 7A, B**, *P* < 0.05). SY promoted HO-1 expression in a dose-dependent manner. After 10, 50, and 100 mg/L SY intervention, the mRNA levels of HO-1 obviously increased to 1.7-, 2.4-, and 3.3-folds of that in H_2_O_2_ treated group (**Figure 7C**, *P* < 0.05). Besides, HSYA also increased HO-1 mRNA levels to 1.4-folds of H_2_O_2_ treated group (**Figure 7F**, *P* < 0.05).

## Discussion

*Carthamus tinctorius L.* is a traditional herbal medicine with multifunctional applications, which has been used in the treatments of cerebrovascular diseases, cardiovascular diseases, and gynecological diseases (Zhang et al., 2016). Modern pharmacological studies have also shown that SY and HSYA, the active components of *Carthamus tinctorius L.*, possess many pharmacological effects (Zhang et al., 2016). In our previous study, SY has been demonstrated with the anti-obesity effects. Similarly, our present study also discovered that SY and its main component HSYA could decrease body weight gain and fat mass, and improve glucose metabolism and liver function in DIO mice, implying a role in alleviating obesity. Preliminary mechanistic investigation showed that SY and HSYA treatment obviously increased the antioxidant enzymes expression in liver and adipose tissue of DIO mice. Further cellular experiments performed in H_2_O_2_-induced oxidative stress HepG2 cells and adipocytes showed that this antioxidant effects of SY and HSYA in DIO mice was achieved by directly increase the expression of antioxidant factor Nrf2 and antioxidant enzymes in HepG2 cells and adipocytes with oxidative stress damage. Taken together, SY and its main component HSYA could alleviate diet-induced obesity in mice, which might be associated with the increased expression of antioxidant enzymes in liver, adipose tissue, and cells.

Consistent with our results, other researchers have also demonstrated the anti-obesity effects of SY and HSYA. It was reported by Bao et al. that SY could reduce body weight and blood lipid levels in the mice fed with HFD (Bao et al., 2015). Liu et al. also found that HSYA could reduce body weight, fat accumulation, and insulin resistance in the HFD-fed mice (Liu et al., 2018). Besides, the promoting effects of SY and HSYA on the expression of Nrf2 and antioxidant enzymes have also been reported in pheochromocytoma cells, HepG2 cells, and cardiomyocytes (Wang et al., 2009; Liu et al., 2012; Ma et al., 2016). Safflower yellow B was found to significantly increase SOD and glutathione peroxidase activities in the H_2_O_2_-injured pheochromocytoma PC12 cells (Wang et al., 2009), and obviously increase the expression of Nrf2, HO-1, and Nqo1 in HepG2 cells with H_2_O_2_-induced oxidative damage (Ma et al., 2016). HSYA was also found to upregulate the expression and activities of HO-1 through the PI3K/Akt/Nrf2 pathway in H9c2 cardiomyocytes (Liu et al., 2012).

In the present study, the anti-obesity effects of SY and HSYA might be associated with the increase expression of antioxidant enzymes. Patients with overweight or obesity have lower antioxidant capacity (Chrysohoou et al., 2007). The activities of antioxidant enzymes such as glutathione peroxidase and SOD in individuals with obesity are significantly lower than healthy people (Ozata et al., 2002). Similarly, obese mice or rats also showed lower antioxidant capacity (Bełtowski et al., 2000; Su et al., 2016), as well as decreased expression of antioxidant enzymes in the adipose tissue (Illesca et al., 2019). However, the regulation of antioxidative enzymes in obesity is complicated. Some literatures also demonstrate that antioxidative enzymes are induced in obesity. It has been reported that SOD activity was higher in obese children compared with normal-weight controls as a consequence of cell adaptation to the increased radical production in obesity (Sfar et al., 2013). There was also significant increase in the SOD levels in the liver, adipose tissues, kidney, testis, muscle, and plasma of obese ob/ob mice (Nakao et al., 2000). Obese Zucker rats have also been found to have increased SOD activity in the myocardial tissue (Vincent et al., 2001). In our current study, the expression of the antioxidant enzymes SOD1 and GCLC in the adipose tissue was significantly decreased in DIO mice. The expression of antioxidant enzymes in adipose tissue could alleviate oxidative stress which is associated with the dysfunction of adipose tissue (Bluher, 2009; Manna and Jain, 2015). Nrf2 is a critical transcription factor that regulates the expression of many antioxidant enzymes, including SOD1, HO-1, GCLC, Nqo1, and CAT (Zhang et al., 2015). Pharmacological activation of Nrf2 has been reported to alleviate obesity and insulin resistance in mice. CDDO-Imidazolide, the synthetic activator of Nrf2 signaling, has been found to prevent high fat diet-induced increases in body weight, fat mass, and hepatic lipid accumulation in C57BL/6J mice (Shin et al., 2009). In the present study, the treatment of SY and HSYA could increase the expression of Nrf2 and antioxidant enzymes in adipose tissue and 3T3-L1 adipocytes, which might contribute to the decrease of fat mass and the improvement of glucose metabolism in DIO mice. To our knowledge, this is the first time that SY and HSYA have been found to directly increase the expression of antioxidant enzymes in adipose tissue, which might help to discover the novel mechanism of its anti-obesity effect.

In addition, NAFLD is a metabolic complication of obesity. Fat accumulation in the hepatocytes exposes liver to oxidative stress, which appears as the most important pathological event during NAFLD development (Spahis et al., 2017). Antioxidants, such as polyphenols and carotenoids, have been proposed as a novel therapeutic approach for NAFLD (Ferramosca et al., 2017). In the present study, the treatment of SY and HSYA is able to decrease serum ALT levels, and reduce lipid droplets accumulation in the liver of DIO mice, indicating the protective effect of SY and HSYA against HFD-induced NAFLD. Moreover, SY and HSYA enhanced SOD activities and increased the mRNA levels of antioxidant enzymes in the liver of DIO mice, suggesting that the antioxidant effect of SY and HSYA may play a role in the mechanism of the hepatoprotective effects.

It is well known that food intake and energy expenditure are the counterbalance factors that determine the body weight change (Piaggi et al., 2018). In the present study, the mice treated with SY or HSYA have a decreased body weight gain, but have no change in food intake. Therefore, it is possible that there might be an increase in the energy expenditure of mice. The effects of SY or HSYA on the basal metabolic rate and physical activity of mice need to be investigated in the future. Besides, skeletal muscle can strongly influence whole-body glucose homeostasis and insulin sensitivity (Wu and Ballantyne, 2017). The potential role of skeletal muscle on mediating the improvement of glucose metabolism and the reduction of insulin resistance after SY and HSYA treatment also remains to be explored in the future study. In addition, it is generally known that reducing body weight and fat mass could effectively improve insulin sensitivity and decrease blood glucose levels (Grams and Garvey, 2015; Pourhassan et al., 2017; Vink et al., 2017). Therefore, it is possible that the beneficial effects of SY and HSYA on improving metabolism in the present study result from the subsequent effects of the decrease of body weight gain and fat mass. The experiment about the short-term effects of SY and HSYA treatments on the metabolic parameters of DIO mice before presenting significant reduction in the body weight gain needs to be done in the future to further clarify the issues.

In conclusion, SY and its main component HSYA play a role in alleviating diet-induced obesity in mice, which might be associated with the increased expression of antioxidant enzymes in liver and adipose tissue. These findings might contribute to develop SY and HSYA as new drugs to tackle obesity. However, further studies for the detailed mechanism, and experiments on the effects of SY and HSYA on patients with obesity, are still needed.

## Data Availability Statement

All datasets generated for this study are included in the article/**Supplementary Material**.

## Ethics Statement

The animal experiment protocols were approved by the ethics committee of Peking Union Medical College Hospital.

## Author Contributions

KY performed the cell experiments and molecular biology experiments, analyzed the data, and wrote the primary manuscript. XW performed the animal experiments. HP, LW, and HY helped to design and perform the experiments. ML helped to analyze the data. FG and HZ designed the experiment, supervised all experiments, and revised the primary manuscript.

## Funding

The study was supported by grants from the Beijing Natural Science Foundation (Nos. 7182130, 7082079 for FG), the National Natural Science Foundation of China (Nos. 81370898 for FG, Nos. 81471024 for HZ), the National Key Program of Clinical Science (WBYZ2011-873 for FG and HZ), PUMCH Foundation (2013-020 for FG), and the Innovation fund for postgraduate students of Peking Union Medical College (2018-1002-01-04 for KY).

## Conflict of Interest

The authors declare that the research was conducted in the absence of any commercial or financial relationships that could be construed as a potential conflict of interest.

## Supplementary Material

Figure S1 Effects of SY and HSYA on the body weight, body weight gain and food intake of mice by intragastric administration. Mice were intragastric administrated with 250 mg/kg/d SY or HSYA. Body weight (A) was recorded, and the body weight gains (B) were calculated four weeks after administration. Food intake (C) was also recorded. The data are represented as the mean ± SD. * P<0.05 vs. SF, # P<0.05 vs. HFD. (n=10 in each group)

Figure S2 Oil Red O staining of liver tissue. The liver tissue (stored at -80 °C) was unfrozen slowly and fixed in 4% paraformaldehyde fix solution (AR1068, Boster Biological Technology Co., Ltd., Wuhan, China). Frozen tissue sections (20 μm) were stained with Oil Red O by standard protocols. Images of the representative sections in the SF-Saline group (A), SF-SY group (B), HFD-Saline group (C), HFD-SY group (D) and HFD-HSYA group (E) at 100× magnification (scale bars, 50 μm) were obtained using a biological microscope (CX31, Olympus, Japan).

Figure S3 Effects of SY and HSYA on serum TNFα levels. Serum levels of tumor necrosis factor α (TNFα) were measured by a commercial ELISA kit (MEA133Mu, Wuhan USCN Business Co., Ltd., Wuhan, China) according to the manufacturer’s instruction. The intra-assay coefficient of variation was 4.3%. The data are represented as the mean ± SD. (n=9 in the SF-Saline group, n=8 in the SF-SY group, n=10 in the HFD-Saline group, n=9 in the HFD-SY group, n=9 in the HFD-HSYA group).

Figure S4 Effects of SY and HSYA on the expression of Nrf2 and antioxidant enzymes in the subcutaneous adipose tissues of the mice. Mice were intraperitoneally injected with 200 mg/kg/d SY or HSYA for ten weeks. The subcutaneous adipose tissue was obtained and total RNA was extracted. The mRNA levels of nuclear factor erythroid 2-related factor 2 (Nrf2) and antioxidant enzymes, including superoxide dismutase 1 (SOD1), heme oxygenase-1 (HO–1), glutamate-cysteine ligase catalytic subunit (GCLC) and NAD(P)H dehydrogenase (quinone 1) (Nqo1) were determined by RT-qPCR analysis (A–E). The data are represented as the mean ± SD. * P<0.05 vs. SF-Saline. (n=8 in each group).

Figure S5 Cell viability evaluated by MTT assay. HepG2 cells (A) and the differentiated 3T3-L1 adipocytes (B) were seeded in 96-well plates. After 24 hours, cells were treated with 0~4000 μM hydrogen peroxide (H2O2) solution for 24h. Cell viability was then evaluated by MTT assay using the MTT Cell Proliferation and Cytotoxicity Assay Kit (Beyotime, Shanghai, China). The data are represented as the mean ± SD of six separate wells. * p<0.05 vs. control (treated with 0 µM H2O2).
