# Supplementary material for: Safflower Yellow and Its Main Component HSYA Alleviate Diet-Induced Obesity in Mice: Possible Involvement of the Increased Antioxidant Enzymes in Liver and Adipose Tissue
Source: Front Pharmacol. 2020 Apr 21;11:482. doi: 10.3389/fphar.2020.00482 (PMC7186386; doi:10.3389/fphar.2020.00482)
Supplement: Supplementary file 1 [file DataSheet_1.pdf]

Supplementary Material

The HPLC analysis of safflower yellow (SY)

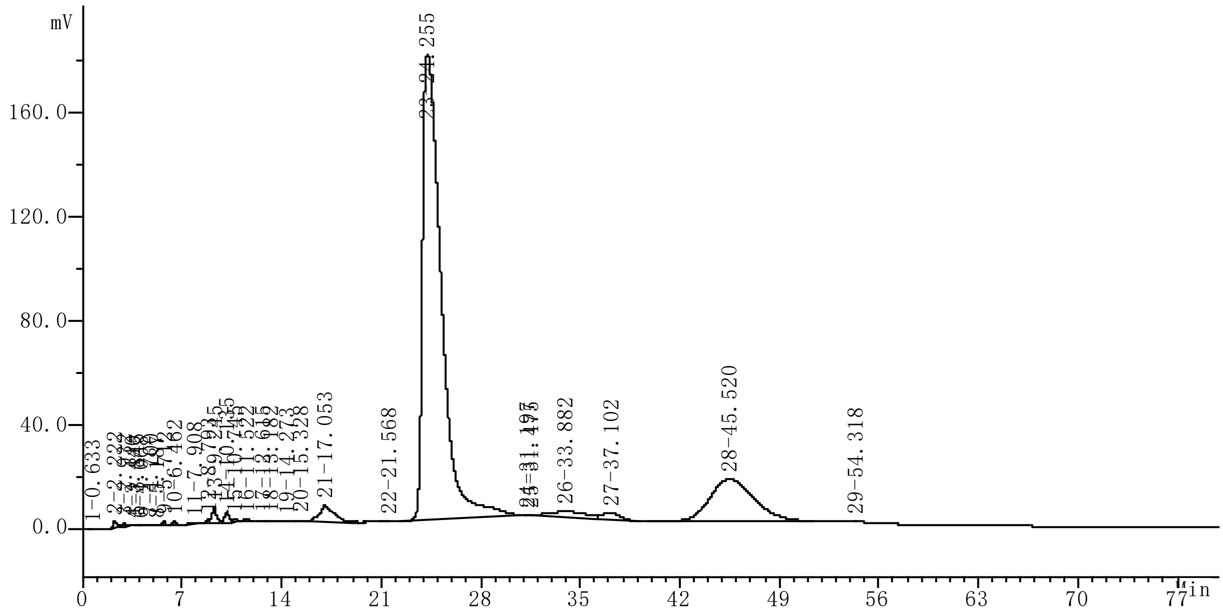

Peak	Ret time [min]	Area	Area %
1	0.633	105	0.0005
2	2.222	41548	0.1905
3	2.932	17356	0.0796
4	3.440	5737	0.0263
5	3.845	1780	0.0082
6	4.068	2138	0.0098
7	4.700	5960	0.0273
8	5.187	242	0.0011
9	5.712	17332	0.0795
10	6.462	23284	0.1068
11	7.908	15506	0.0711
12	8.793	27904	0.1280
13	9.245	145203	0.6659
14	10.135	99283	0.4553
15	10.745	29326	0.1345
16	11.522	39651	0.1818
17	12.615	9018	0.0414
18	13.182	14038	0.0644
19	14.273	12844	0.0589
20	15.328	14020	0.0643
21	17.053	460358	2.1111
22	21.568	3608	0.0165
23	24.255	16476815	75.5602
24	31.197	375	0.0017
25	31.475	2656	0.0122
26	33.882	456737	2.0945
27	37.102	245431	1.1255
28	45.520	3637811	16.6824
29	54.318	158	0.0007
Total		21806222	100
