# Supplementary figures and images for: Safflower Yellow and Its Main Component HSYA Alleviate Diet-Induced Obesity in Mice: Possible Involvement of the Increased Antioxidant Enzymes in Liver and Adipose Tissue
Source: Front Pharmacol. 2020 Apr 21;11:482. doi: 10.3389/fphar.2020.00482 (PMC7186386; doi:10.3389/fphar.2020.00482)

Supplementary Material

The structure and HPLC analysis of HSYA

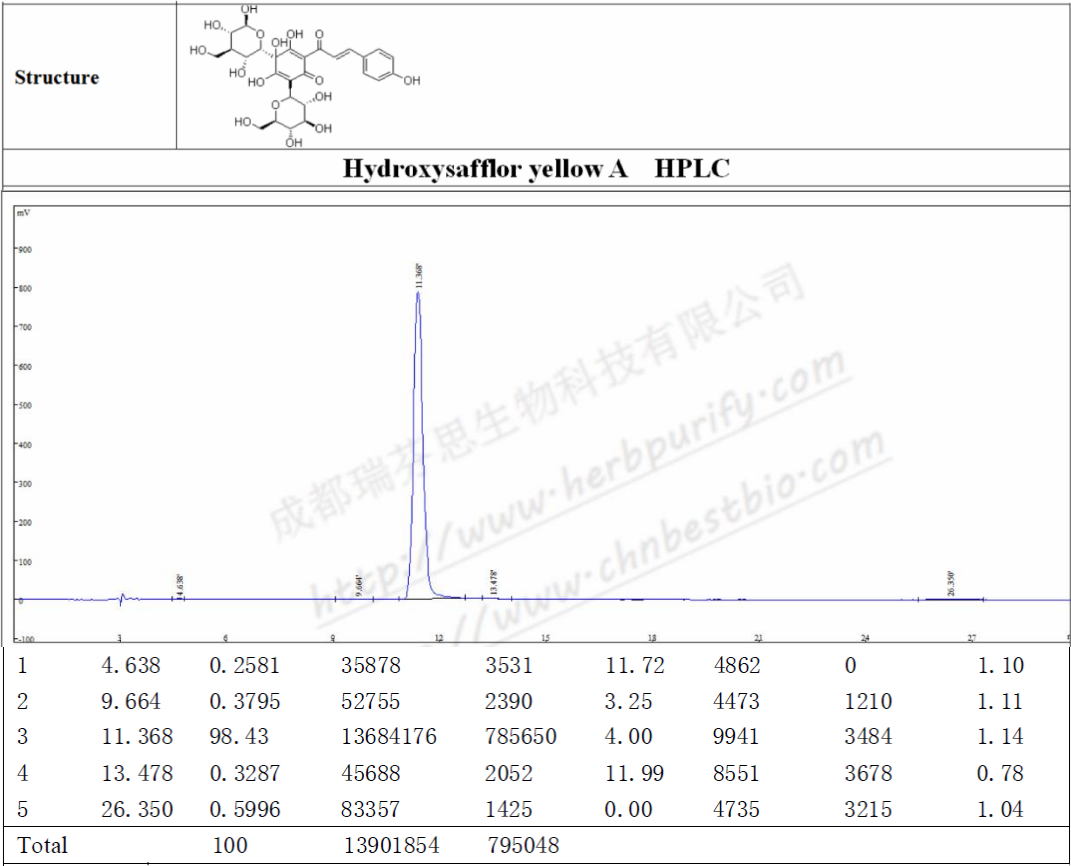

Supplement: Supplementary file 2 [file DataSheet_2.pdf]

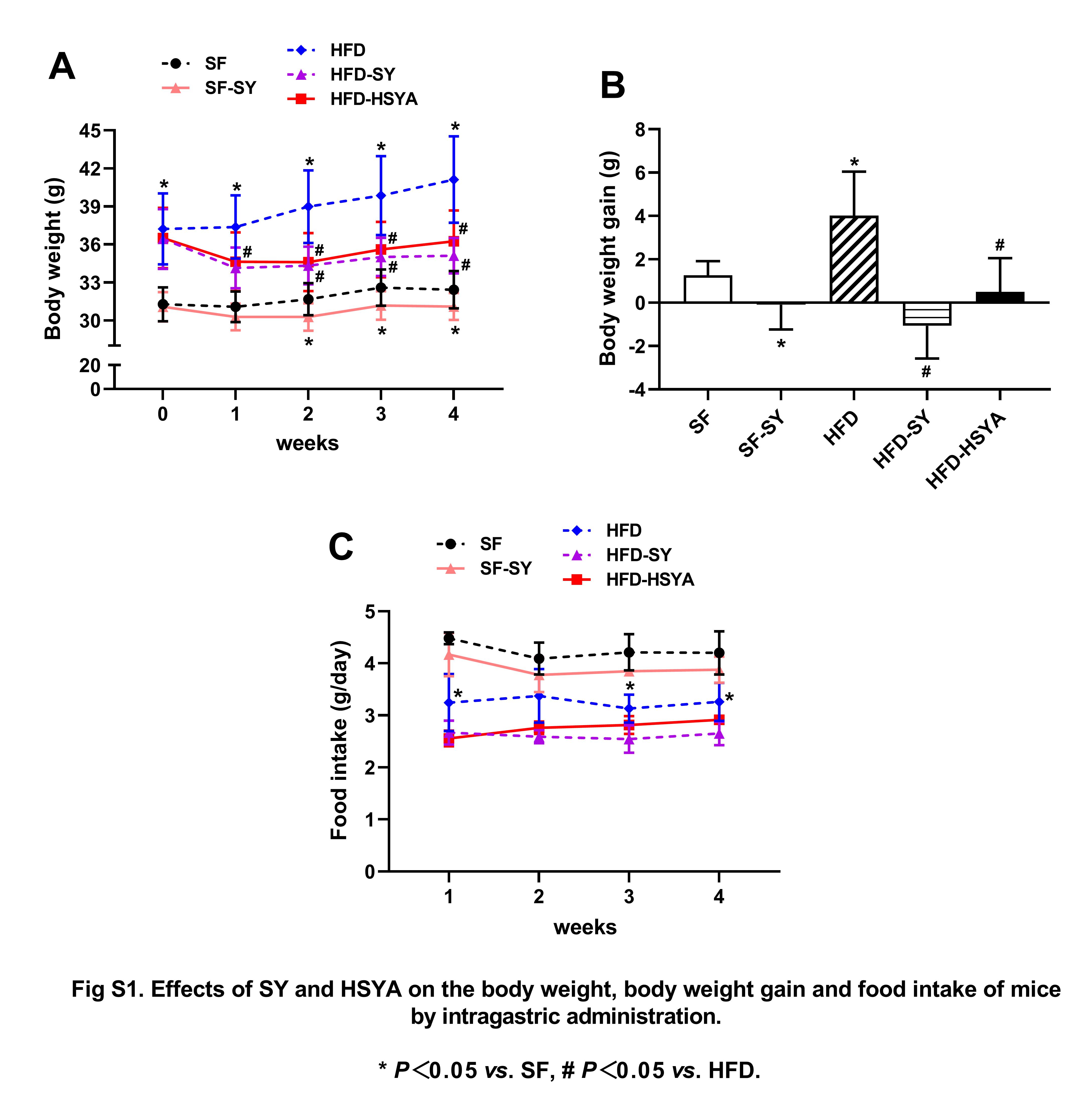

Supplement: Figure S1 — Effects of SY and HSYA on the body weight, body weight gain and food intake of mice by intragastric administration. Mice were intragastric administrated with 250 mg/kg/d SY or HSYA. Body weight (A) was recorded, and the body weight gains (B) were calculated four weeks after administration. Food intake (C) was also recorded. The data are represented as the mean ± SD. * P<0.05 vs. SF, # P<0.05 vs. HFD. (n=10 in each group) [file Image_1.jpeg]

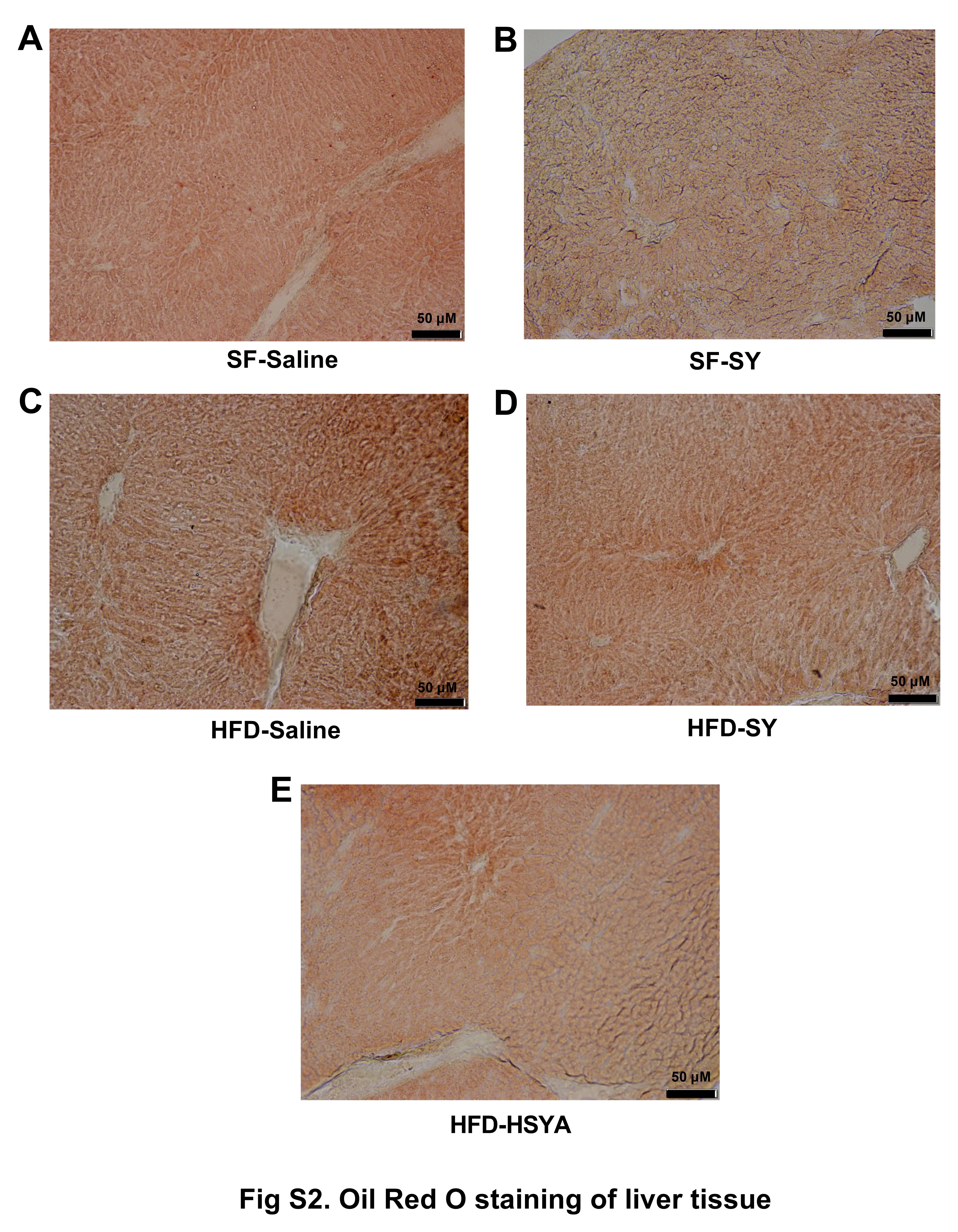

Supplement: Figure S2 — Oil Red O staining of liver tissue. The liver tissue (stored at -80 °C) was unfrozen slowly and fixed in 4% paraformaldehyde fix solution (AR1068, Boster Biological Technology Co., Ltd., Wuhan, China). Frozen tissue sections (20 μm) were stained with Oil Red O by standard protocols. Images of the representative sections in the SF-Saline group (A), SF-SY group (B), HFD-Saline group (C), HFD-SY group (D) and HFD-HSYA group (E) at 100× magnification (scale bars, 50 μm) were obtained using a biological microscope (CX31, Olympus, Japan). [file Image_2.jpeg]

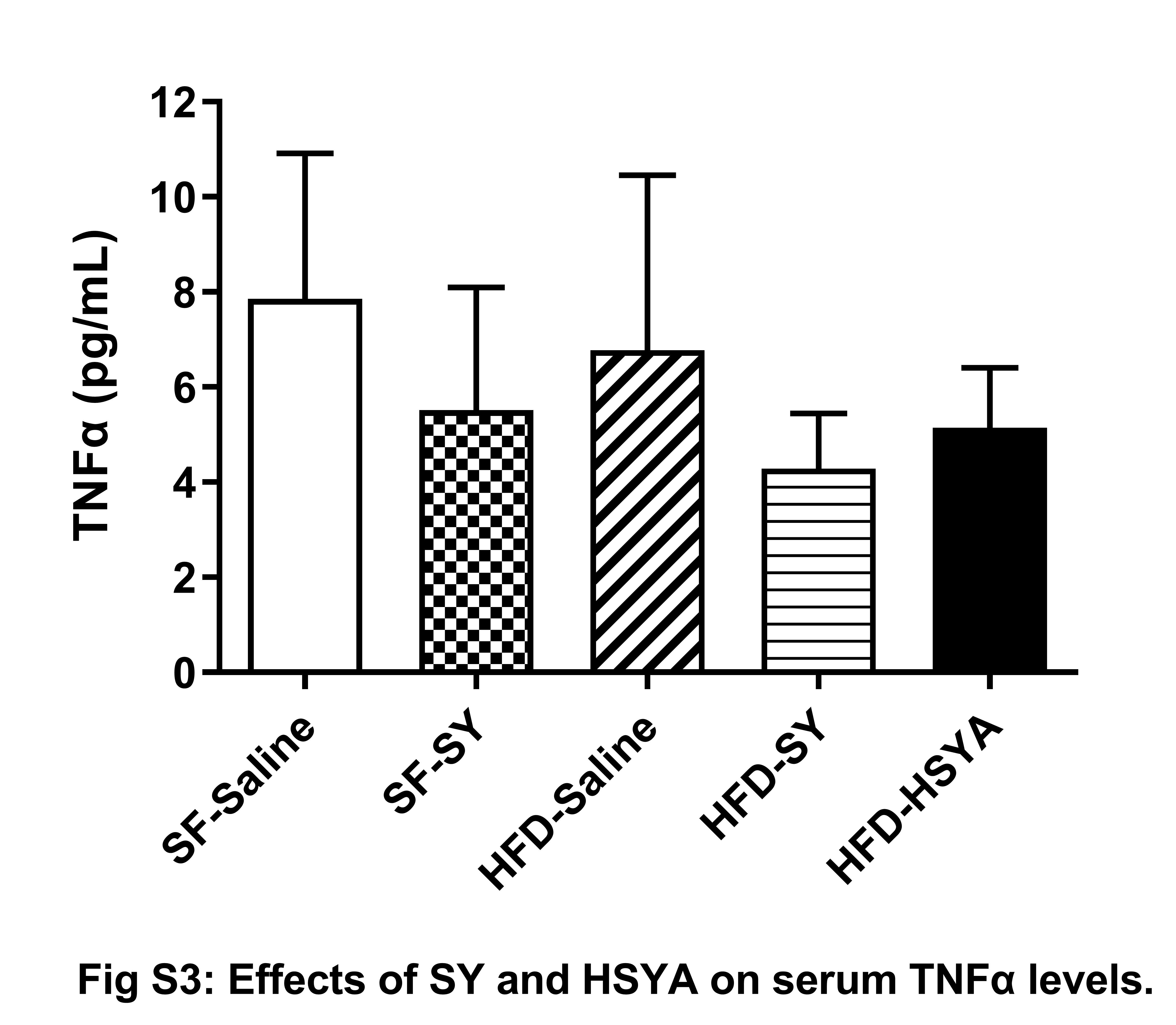

Supplement: Figure S3 — Effects of SY and HSYA on serum TNFα levels. Serum levels of tumor necrosis factor α (TNFα) were measured by a commercial ELISA kit (MEA133Mu, Wuhan USCN Business Co., Ltd., Wuhan, China) according to the manufacturer’s instruction. The intra-assay coefficient of variation was 4.3%. The data are represented as the mean ± SD. (n=9 in the SF-Saline group, n=8 in the SF-SY group, n=10 in the HFD-Saline group, n=9 in the HFD-SY group, n=9 in the HFD-HSYA group). [file Image_3.jpeg]

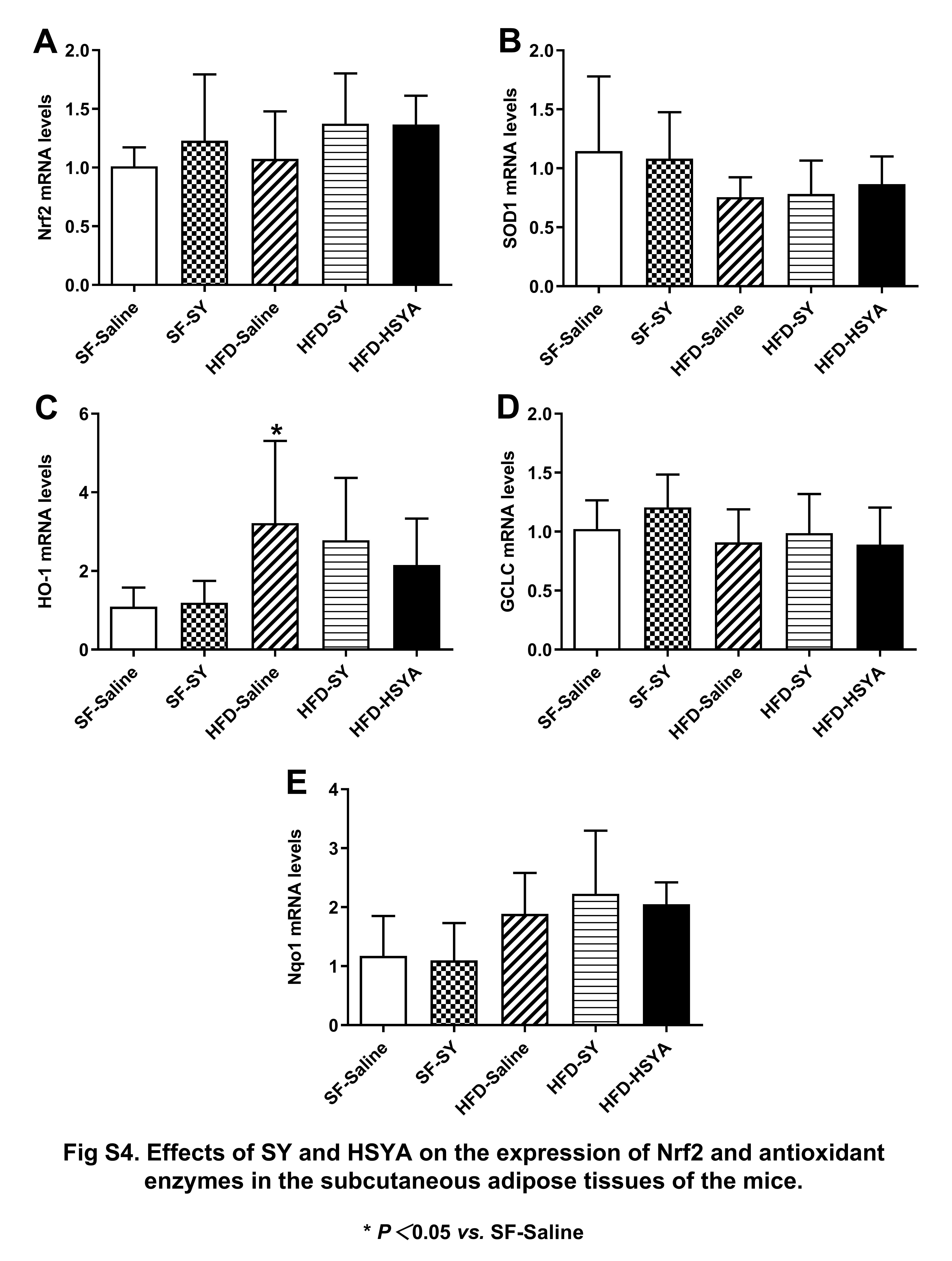

Supplement: Figure S4 — Effects of SY and HSYA on the expression of Nrf2 and antioxidant enzymes in the subcutaneous adipose tissues of the mice. Mice were intraperitoneally injected with 200 mg/kg/d SY or HSYA for ten weeks. The subcutaneous adipose tissue was obtained and total RNA was extracted. The mRNA levels of nuclear factor erythroid 2-related factor 2 (Nrf2) and antioxidant enzymes, including superoxide dismutase 1 (SOD1), heme oxygenase-1 (HO–1), glutamate-cysteine ligase catalytic subunit (GCLC) and NAD(P)H dehydrogenase (quinone 1) (Nqo1) were determined by RT-qPCR analysis (A–E). The data are represented as the mean ± SD. * P<0.05 vs. SF-Saline. (n=8 in each group). [file Image_4.jpeg]

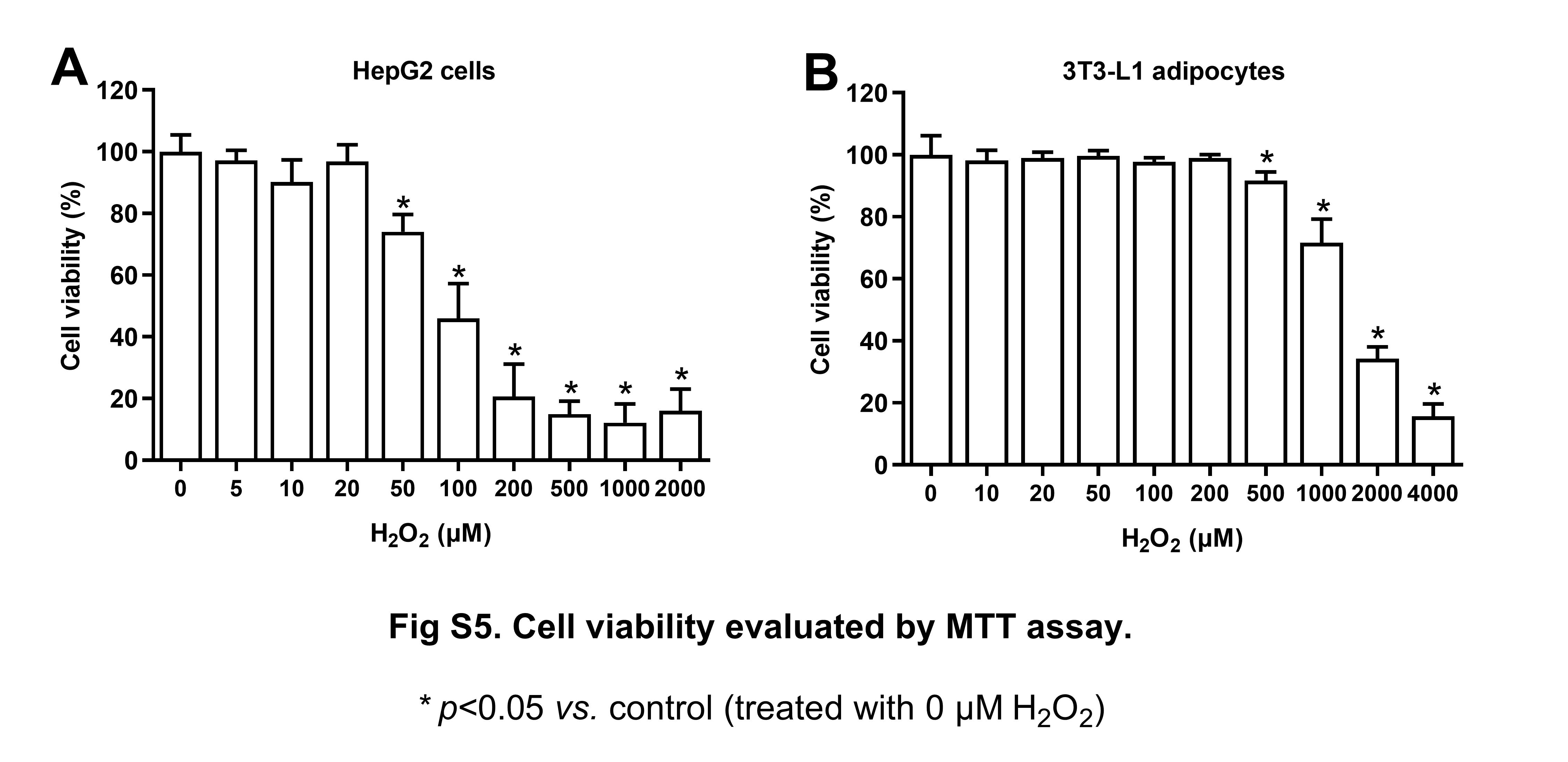

Supplement: Figure S5 — Cell viability evaluated by MTT assay. HepG2 cells (A) and the differentiated 3T3-L1 adipocytes (B) were seeded in 96-well plates. After 24 hours, cells were treated with 0~4000 μM hydrogen peroxide (H2O2) solution for 24h. Cell viability was then evaluated by MTT assay using the MTT Cell Proliferation and Cytotoxicity Assay Kit (Beyotime, Shanghai, China). The data are represented as the mean ± SD of six separate wells. * p<0.05 vs. control (treated with 0 µM H2O2). [file Image_5.jpeg]
