# Supplementary material for: Safflower Yellow and Its Main Component HSYA Alleviate Diet-Induced Obesity in Mice: Possible Involvement of the Increased Antioxidant Enzymes in Liver and Adipose Tissue
Source: Front Pharmacol. 2020 Apr 21;11:482. doi: 10.3389/fphar.2020.00482 (PMC7186386; doi:10.3389/fphar.2020.00482)
Supplement: Supplementary file 3 [file Table_1.docx]

# Supplementary Material

# Table S1: The compositions of the experimental diets

| **Component** | **Standard food (g)** | **High fat diet (g)** |
| --- | --- | --- |
| **Ingredients (g)** |  |  |
| Casein | 189.58 | 233.06 |
| Corn starch | 298.59 | 84.83 |
| Sucrose | 331.77 | 201.36 |
| Dextrose | 33.18 | 116.53 |
| Cellulose | 47.40 | 58.26 |
| AIN-93 Vitamin mixture | 9.48 | 11.56 |
| AIN-93 Mineral mixture | 9.48 | 11.65 |
| Soybean oil | 23.70 | 29.13 |
| Lard | 18.96 | 206.84 |
| L-cystine | 2.84 | 3.50 |
| Choline Bitartrate | 1.90 | 2.33 |
| Calcium hydrophosphate | 12.32 | 15.15 |
| Calcium carbonate | 5.21 | 6.41 |
| Potassium citrate | 15.64 | 19.23 |
| TBHQ (tert-butylhydroquinone) | 0.047 | 0.058 |
| **Energy (%)** |  |  |
| Protein | 20 | 20 |
| Lipid | 10 | 45 |
| Carbohydrate | 70 | 35 |
