# Supplementary material for: Safflower Yellow and Its Main Component HSYA Alleviate Diet-Induced Obesity in Mice: Possible Involvement of the Increased Antioxidant Enzymes in Liver and Adipose Tissue
Source: Front Pharmacol. 2020 Apr 21;11:482. doi: 10.3389/fphar.2020.00482 (PMC7186386; doi:10.3389/fphar.2020.00482)
Supplement: Supplementary file 4 [file Table_2.docx]

# Supplementary Material

# Table S2: Primers for RT-qPCR

|  | **Forward primer (5’-3’)** | **Reverse primer (5’-3’)** |
| --- | --- | --- |
| m-PPIA | GCTGGACCAAACACAAACGG | TCCTGGACCCAAAACGCTC |
| m-Nrf2 | AAGCACAGCCAGCACATTC | GGATTCACGCATAGGAGCAC |
| m-SOD1 | TGTGGAGTGATTGGGATTGC | CAGTTTAATGGTTTGAGGGTAGC |
| m-GCLC | GGTGTCCGCTCTTCCATTAC | GCCTGTCAATCTGCTCCTG |
| m-Nqo1 | TTCAGAGAAGACATCATTCAACTAC | TCATAGCATAGAGGTCAGATTCG |
| m-CAT | TGCGGACATTCTACACAAAGG | TTGCGTTCTTAGGCTTCTCAG |
| m-HO-1 | GCTAAGACCGCCTTCCTG | AAGTGACGCCATCTGTGAG |
| h-GAPDH | ACAACTTTGGTATCGTGGAAGG | GCCATCACGCCACAGTTTC |
| h-Nrf2 | TCAGCGACGGAAAGAGTATGA | CCACTGGTTTCTGACTGGATGT |
| h-GCLC | GGAGACCAGAGTATGGGAGTT | CCGGCGTTTTCGCATGTTG |
| h-Nqo1 | GAAGAGCACTGATCGTACTGGC | GGATACTGAAAGTTCGCAGGG |
| h-SOD1 | GGTGGGCCAAAGGATGAAGAG | CCACAAGCCAAACGACTTCC |
| h-CAT | TGGAGCTGGTAACCCAGTAGG | CCTTTGCCTTGGAGTATTTGGTA |

m: mouse; h: human
